# Effect of coronal plane acetabular correction on joint contact pressure in Periacetabular osteotomy: a finite-element analysis

**DOI:** 10.1186/s12891-022-05005-5

**Published:** 2022-01-14

**Authors:** Kenji Kitamura, Masanori Fujii, Miho Iwamoto, Satoshi Ikemura, Satoshi Hamai, Goro Motomura, Yasuharu Nakashima

**Affiliations:** 1 Department of Orthopaedic Surgery, Graduate School of Medical Sciences, Kyushu University, 3-1-1 Maidashi, Higashi-ku, Fukuoka, 812-8582 Japan; 2 Department of Orthopaedic Surgery, Faculty of Medicine, Saga University, 5-1-1 Nabeshima, Saga, 849-8501 Japan

**Keywords:** Hip dysplasia, Periacetabular osteotomy, Finite-element analysis, Joint contact pressure

## Abstract

**Background:**

The ideal acetabular position for optimizing hip joint biomechanics in periacetabular osteotomy (PAO) remains unclear. We aimed to determine the relationship between acetabular correction in the coronal plane and joint contact pressure (CP) and identify morphological factors associated with residual abnormal CP after correction.

**Methods:**

Using CT images from 44 patients with hip dysplasia, we performed three patterns of virtual PAOs on patient-specific 3D hip models; the acetabulum was rotated laterally to the lateral center-edge angles (LCEA) of 30°, 35°, and 40°. Finite-element analysis was used to calculate the CP of the acetabular cartilage during a single-leg stance.

**Results:**

Coronal correction to the LCEA of 30° decreased the median maximum CP 0.5-fold compared to preoperatively (*p* <  0.001). Additional correction to the LCEA of 40° further decreased CP in 15 hips (34%) but conversely increased CP in 29 hips (66%). The increase in CP was associated with greater preoperative extrusion index (*p* = 0.030) and roundness index (*p* = 0.038). Overall, virtual PAO failed to normalize CP in 11 hips (25%), and a small anterior wall index (*p* = 0.049) and a large roundness index (*p* = 0.003) were associated with residual abnormal CP.

**Conclusions:**

The degree of acetabular correction in the coronal plane where CP is minimized varied among patients. Coronal plane correction alone failed to normalize CP in 25% of patients in this study. In patients with an anterior acetabular deficiency (anterior wall index < 0.21) and an aspherical femoral head (roundness index > 53.2%), coronal plane correction alone may not normalize CP. Further studies are needed to clarify the effectiveness of multiplanar correction, including in the sagittal and axial planes, in optimizing the hip joint’s contact mechanics.

## Background

Periacetabular osteotomy (PAO) is an established surgical treatment for young adults with symptomatic hip dysplasia that improves the containment of the femoral head, structural instability, and abnormal cartilage loading via three-dimensional (3D) acetabular correction [1–3]. The goal of PAO is to correct hip pathomechanics, relieve pain, maintain or improve patients’ activity and quality of life, and prevent or delay secondary osteoarthritis (OA). While mid- to long-term studies have reported generally favorable outcomes of PAO, poor prognostic factors include older age, advanced OA grade, joint incongruity, and suboptimal acetabular correction such as under- or over-coverage [4–8]. Several studies have suggested that the prognosis of PAO can be optimized by adjusting the lateral center-edge angle (LCEA) between 30° and 40° [5, 9].

Previous finite element (FE) analysis studies have shown that acetabular correction in the coronal plane reduces joint contact pressure (CP) in cases of hip dysplasia [10, 11]; however, it remains unclear what the ideal position should be for each patient in order to optimize the contact mechanics of dysplastic hips. The 3D morphology of the hip joint substantially varies among candidates for PAO [12, 13], and clinical studies have shown that preoperative severe acetabular dysplasia [14] and aspherical femoral heads that lead to joint incongruity [15, 16] compromise joint survivorship following PAO. However, to date, it is not fully understood how preoperative hip morphology influences joint CP after coronal plane correction. In addition, previous FE analysis studies have used a standardized pelvic position based on the anterior pelvic plane (APP) coordinate system, whereas biomechanics-based planning for PAO is recommended to account for patient-specific functional pelvic tilt in the weight-bearing position [17].

Determining the optimal extent of acetabular correction and associated morphological factors from a biomechanical perspective may improve acetabular reorientation during PAO and lead to better clinical outcomes. In this study, we performed virtual PAO using patient-specific FE models in the standing pelvic position as a reference to determine the relationship between the amount of acetabular correction in the coronal plane and joint CP and identify radiographic factors associated with residual abnormal joint CP after correction.

## Methods

### Patients

This retrospective study was approved by our institution’s review board. Ninety-two patients (100 hips) with symptomatic hip dysplasia underwent transposition osteotomy of the acetabulum (TOA) [18] between September 2016 and July 2020. We reviewed preoperative supine and standing anteroposterior (AP) pelvic radiographs and pelvic CT images (matrix: 512 × 512, field of view: 260–696 mm, and slice thickness: 1 or 2 mm) that were taken during preoperative examination. Eighty-eight patients (96 hips) with hip dysplasia, defined as a LCEA < 20° on supine AP pelvic radiographs [19], were included in this study. In patients with bilateral hip dysplasia, the operated-on side was investigated. Exclusion criteria for this study included advanced OA (Tönnis grade [20] ≥ 2) (*n* = 13), major femoral head deformity (*n* = 1), previous surgery on either hip joint (*n* = 15), previous spinal surgery (*n* = 1), and poor-quality images (*n* = 14). Thus, 44 patients (44 hips) were eligible for this study. All patients were female with a mean age of 38.1 ± 10.3 years and a mean LCEA of 9.7 ± 6.8° (Table 1).

**Table 1:** Background data of patients with hip dysplasia (*n* = 44)

Parameters	
Age (years) ^a^	38.1 ± 10.3
Sex ^b^	
Male	0 (0)
Female	44 (100)
Body mass index (kg/m^2^) ^a^	22.3 ± 3.8
Height (cm)	157.5 ± 6.0
Weight (kg)	55.2 ± 8.8
Laterality ^b^	
Right hip	28 (64)
Left hip	16 (36)
Tönnis classification system ^b^	
Grade 0	25 (57)
Grade 1	19 (43)
Lateral center-edge angle (°) ^a^	9.7 ± 6.8

^a^Values are presented as the mean ± SD

^b^Values are presented as the number (%)

### Radiographic and CT evaluations

The following morphological parameters were measured from standing AP pelvic radiographs: LCEA, medial center-edge angle (MCEA), acetabular arc, Tönnis angle, sharp angle, extrusion index, crossover sign, posterior wall sign, anterior and posterior wall indexes, roundness index of the femoral head, and femoro-epiphyseal acetabular roof index (Table 2) [15, 21, 22]. The sagittal pelvic tilt in the standing position was measured using the 2D-3D matching technique described in a previous study [23]. Briefly, 3D Template software (Kyocera Medical Corporation, Osaka, Japan) was used to reproduce the sagittal pelvic tilt seen on the standing AP radiograph on the digitally reconstructed radiographs created from CT images by matching the vertical-to-horizontal ratio of the pelvic foramen. Sagittal pelvic tilt was measured as the angle formed by the APP and the vertical axis (APP angle), with positive values representing the anterior tilt of the pelvis (Table 2).

**Table 2:** Measurement parameters

Parameters	
Morphological parameters on supine pelvic radiographs	
Lateral center-edge angle (°) ^a^	11.1 (− 14.8 to 17.4)
Medial center-edge angle (°) ^a^	49.2 ± 8.9
Acetabular arc (°) ^a^	58.3 ± 9.7
Tönnis angle (°) ^a^	22.9 (10.6–34.7)
Sharp angle (°) ^a^	47.8 ± 3.5
Extrusion index (%) ^a^	36.5 (27.1–63.8)
Crossover sign ^b^	13 (29.5)
Posterior wall sign ^b^	33 (75.0)
Anterior wall index ^a^	0.26 (−0.02 to 0.53)
Posterior wall index ^a^	0.87 ± 0.18
Roundness index (%) ^a^	52.4 ± 2.2
Femoro-epiphyseal acetabular roof index ^a^	4.3 (−1.5 to 20.9)
Standing anterior pelvic plane angle (°) ^a^	1.7 ± 6.0

^a^Values are presented as the mean ± SD or the median (range)

^b^Values are presented as a number (%)

### Virtual periacetabular osteotomy

**Fig. 1:**
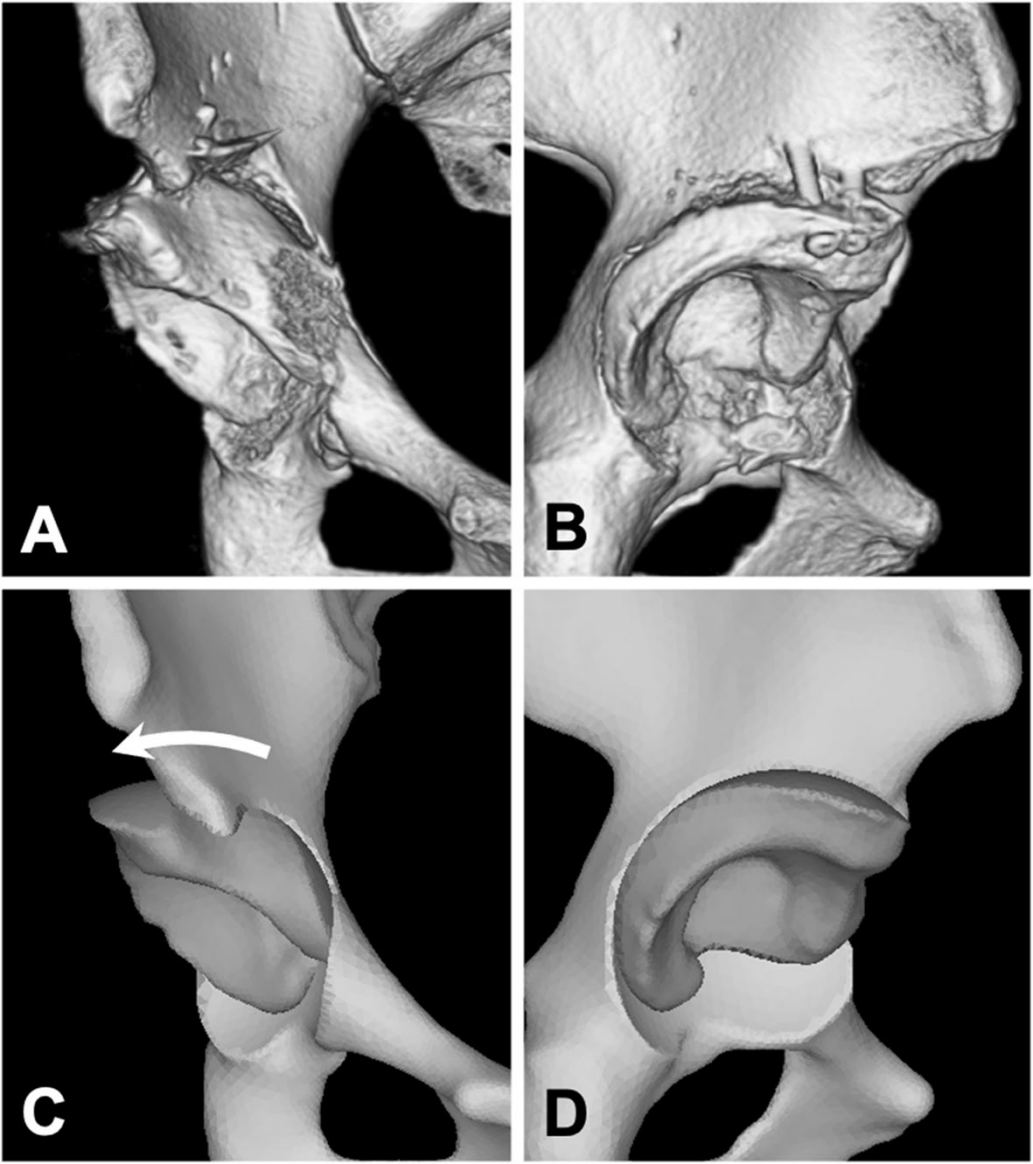
**A** Anteroposterior and **B** lateral views of a computed tomography image of a patient with a right dysplastic hip after transposition osteotomy of the acetabulum (TOA). **C** Anteroposterior and **D** lateral views of the 3D surface model created from CT images of the same patient after virtual periacetabular osteotomy mimicking TOA

**Fig. 2:**
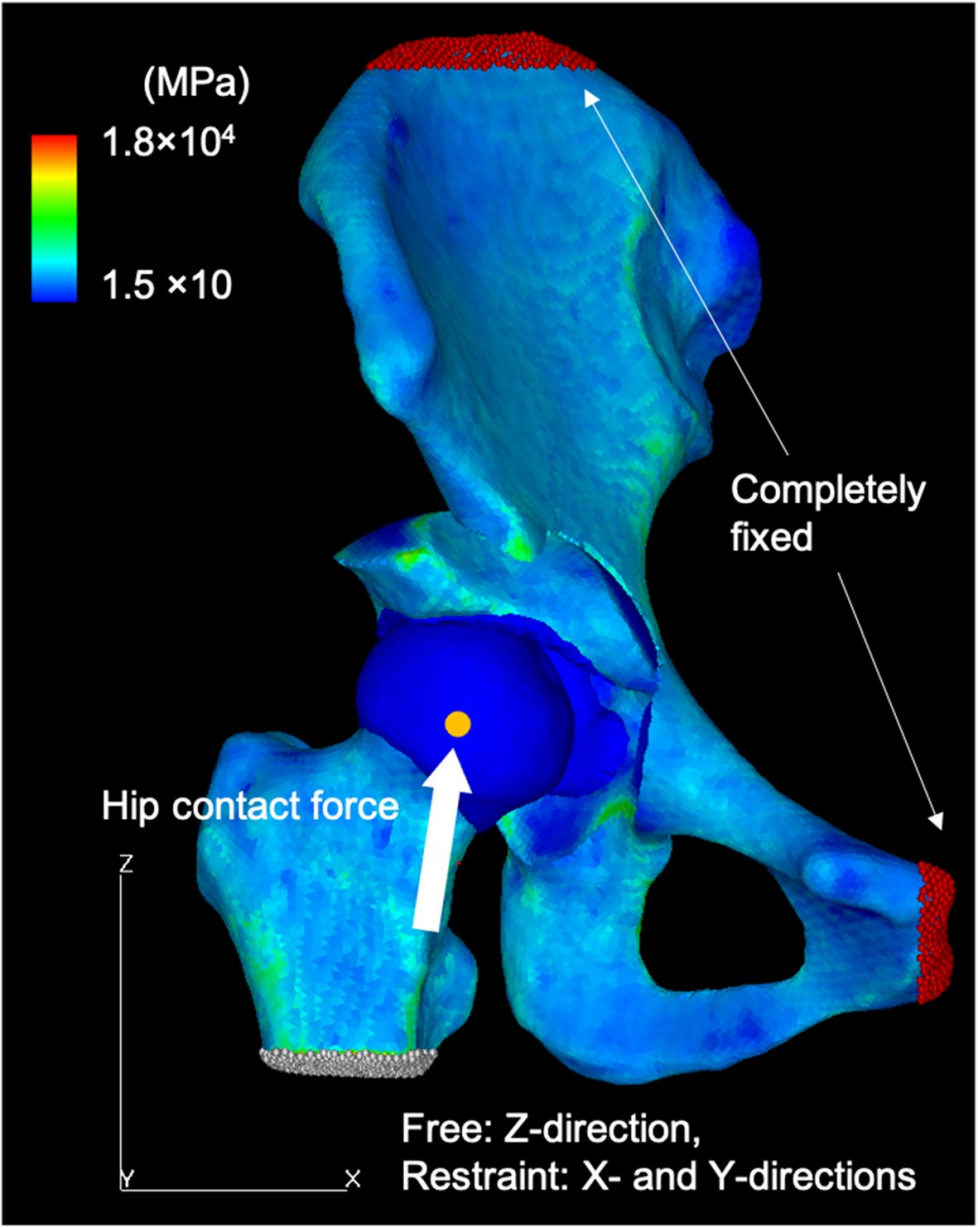
A representative finite-element model of a dysplastic hip after virtual periacetabular osteotomy, with the distribution of the elastic modulus. The bone model was produced with a 2-mm tetrahedral element and a 0.4-mm triangular shell element on its surface. The cartilage of the acetabulum and femoral heads was created with a constant thickness of 1.8 mm and discretized using a locally refined 0.5-mm to 2.0-mm tetrahedral element in the weightbearing region of the acetabular cartilage. To visualize the contact pressure exerted on the acetabular cartilage, three-nodal point shell elements with a thickness of 0.0005 mm were placed on its surface. The loading scenario was based on a single-leg stance, with the hip contact force acting on the nodal point at the femoral head center. During loading, the iliac crest and pubic area were completely fixed, while the distal femur was kept free only in the Z-axis while restrained in the X- and Y-axes. Tied- and sliding-contact constraints were set on the cartilage-to-bone and cartilage-to-cartilage interfaces, respectively. The acetabular fragment was reconnected to the pelvis through a tied contact to simulate a complete bony union. The frictional shear stress between the contacting articular surfaces was ignored

3D surface models of the hemipelvis, proximal femur, and articular cartilage were created using the Mechanical Finder version 10 (Research Centre for Computational Mechanics Inc., Tokyo, Japan) as described in previous studies [17, 24]. Specifically, the bone was modeled using 2-mm tetrahedral elements, whereas three-nodal point shell elements with a thickness of 0.4 mm were employed to model the outer surface of the cortical bone. The cartilage of both the acetabulum and the femoral head were modeled with a constant thickness of 1.8 mm [11]. The articular surface was discretized using tetrahedral elements with a thickness of 0.5–2.0 mm, with local refinement in the weight-bearing area of acetabular cartilage. To visualize the contact pressure exerted on the acetabular cartilage, three-nodal point shell elements with a thickness of 0.0005 mm were placed on its surface. Virtual PAO was performed on pelvic surface models in the same manner as TOA, which is characterized by a spherical osteotomy via a lateral approach (Fig. 1) [18]. The radius of the osteotomy line is 40 mm in most female patients in clinical practice; hence, a spherical osteotomy line with a radius of 40 mm centered on the femoral head center was used in virtual PAO [25]. A total of three patterns of virtual PAOs were performed on each pelvis. The acetabular fragment was rotated laterally on the coronal plane so that the LCEA values were 30°, 35°, and 40° on the standing AP pelvic radiograph. Each model was then meshed into a total of approximately 1.4 million FEs and 65,000 shell elements including the bone and cartilage models. (Fig. 2) [17, 24]. The mean numbers of FEs and shell elements did not differ between models before and after the virtual PAO was performed. The heterogeneous distribution of bone mineral densities (ρ in g/cm^3^) was estimated from the Hounsfield units (HU) of each image by assuming a linear relationship between the HU values and bone mineral density [24]. The elastic FE modulus was determined from bone mineral density values using the equations described by Keyak et al. [26]. The Poisson’s ratio of the bone was set at 0.3 [10, 11]. The elastic modulus and Poisson’s ratio of the articular cartilage were set at 15 MPa and 0.45, respectively [10, 11].

### Boundary and loading conditions

Nonlinear contact analyses were performed using the FE models before and after three patterns of virtual PAOs, and the joint contact area and joint CP of the acetabular cartilage were calculated. We defined the acetabular position with the lowest maximum CP among three patterns of virtual PAOs as the optimal position.

During loading, the coordinate system of the pelvis was set to the standing pelvic position and that of the femur was set according to the definitions by the International Society of Biomechanics [27]. Tied- and sliding-contact constraints were set on the cartilage-to-bone and cartilage-to-cartilage interfaces, respectively [24]. The friction coefficient between the articular cartilage surfaces was reportedly very low (0.01–0.02) in the presence of synovial fluid, suggesting that it was reasonable to neglect frictional shear stresses [28]. In the virtual PAO models, the acetabular fragment was reconnected to the pelvis through a tied contact to simulate a complete bony union. The top surfaces of the pelvis and pubic areas were fixed, while the distal femur was constrained to prevent displacement in the X- and Y-axes while being free in the Z-axis. The loading scenario was based on a single-leg stance, with the contact force of the hip acting on the nodes of the femoral head center (Fig. 2) [29]. A consistent weight of 500 N was defined for all patients to avoid the scaling effect of weight on absolute CP values. The total joint contact force was 1158 N, with the components of the X-, Y-, and Z-axes of 150 N, 71 N, and 1146 N, respectively. The loaded nodes were translated along the loading axis until the desired load was achieved.

### Statistical analyses

A paired t-test or Wilcoxon signed-rank test with Bonferroni correction was used to compare continuous parameters before and after virtual PAO, depending on their distribution and homoscedasticity (Shapiro-Wilk W test and F test). Statistical significance was set at *p* <  0.05. The correlation between two continuous parameters was evaluated using Pearson’s or Spearman’s correlation coefficients, as appropriate. Multivariate logistic regression analysis was used to identify the radiographic factors associated with an increase in CP when LCEA was changed from 30° to 40° by virtual PAO. Those variables with *p* <  0.05 were included in a multivariable model to identify the independent influence of each factor. The same statistical method was used to identify the radiographic factors associated with abnormal CP after virtual PAO. To determine the normal range of joint CP, a receiver operating characteristic (ROC) curve was plotted and the sensitivity, specificity, and cut-off values of maximum CP were calculated for the combined cohort of 44 hip dysplasia patients in this study and 16 normal hip subjects in a previous study [17]. Statistical analyses were performed using the JMP® version 15.0 (SAS Institute, Cary, NC, USA).

## Results

### Relationship between coronal plane correction and joint contact pressure

Coronal plane correction of LCEA to 30° (median correction angle, 18.9° [range, 12.6°-44.8°]) increased the mean contact area 1.8-fold (480 mm^2^ vs. 881 mm^2^, *p* <  0.01) and decreased the median maximum CP 0.5-fold (7.4 MPa vs. 3.8 MPa, *p* < 0.001) compared to preoperative values (Table 3). As the LCEA was increased to 35° and 40°, the mean contact area further increased while the median maximum CP was comparable between the three virtual PAOs. The number of hips in the optimal position, i.e., the acetabular position with the lowest maximum CP among three virtual PAOs, was highest at LCEA 30° (55%), followed by LCEA 35° (25%) and LCEA 40° (20%) (Table 3).

**Table 3:** The contact area and the maximum joint contact pressure before and after virtual periacetabular osteotomy

	Contact area (mm^2^), mean ± SD	Maximum CP (MPa), median (range)	Hips with the optimal position, n (%)	Hips with a normal CP (< 4.1 MPa), n (%)
Before virtual PAO	480 ± 144 ^a^	7.4 (4.1–13.9) ^a^		0 (0)
After virtual PAO				
LCEA 30°	881 ± 168 ^b^	3.8 (2.3–6.7)	24 (55)	28 (64)
LCEA 35°	939 ± 190	3.9 (2.3–7.2)	11 (25)	25 (57)
LCEA 40°	982 ± 212	3.8 (2.5–8.1)	9 (20)	23 (52)

*LCEA* lateral center-edge angle, *CP* contact pressure, *PAO* periacetabular osteotomy

^a^*p* < 0.01 versus all virtual PAOs

^b^*p* < 0.01 between all virtual PAOs

**Fig. 3:**
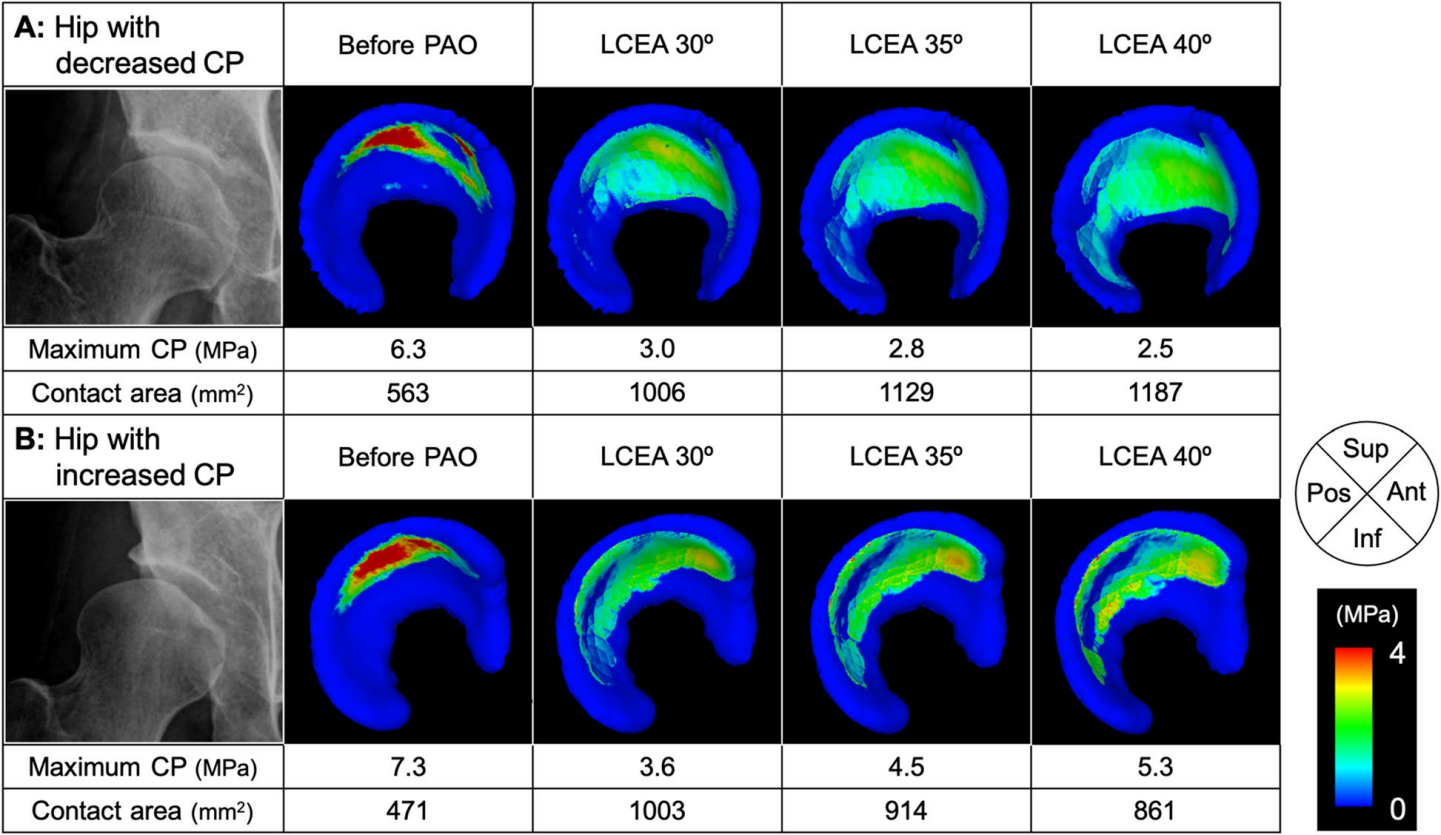
Preoperative anteroposterior pelvic radiographs and distributions of joint contact pressure (CP) on the acetabular cartilage before and after virtual periacetabular osteotomy (PAO) in two representative cases with hip dysplasia (right dysplastic hips). **A** In one case, the maximum CP decreased by increasing the lateral center-edge angle (LCEA) from 30° to 40°, while (**B**) in the other case, the maximum CP increased. Case B had larger extrusion index (47.2 vs. 27.6) and roundness index (53.4 vs. 52.1) than case A. The reference locations of the acetabular cartilage at the standing position are denoted as anterior (Ant), superior (Sup), posterior (Pos), and inferior (Inf)

When comparing the maximum CP between LCEA 30° and 40°, the maximum CP decreased in 15 hips (34%) by increasing the LCEA from 30° to 40°, but conversely increased in 29 hips (66%) (Fig. 3). The change in the maximum CPs from LCEA 30° to 40° was correlated with the LCEA, MCEA, extrusion index, and roundness index on the preoperative radiographs (Table 4). A multivariate logistic regression analysis showed that greater preoperative extrusion index (*p* = 0.030) and roundness index (*p* = 0.038) were independently associated with an increase in maximum CP from LCEA 30° to 40° (Table 5).

**Table 4:** Correlations of preoperative radiographic parameters with change in maximum contact pressure after additional coronal correction

Radiographic parameters	Correlation coefficient ^a^	*p* value
Lateral center-edge angle	−0.34	0.024
Medial center-edge angle	0.37	0.013
Acetabular arc	0.01	0.927
Tönnis angle	0.29	0.056
Sharp angle	0.21	0.175
Extrusion index	0.49	< 0.001
Anterior wall index	−0.12	0.450
Posterior wall index	−0.15	0.323
Roundness index	0.47	0.001
Femoro-epiphyseal acetabular roof index	0.13	0.390

^a^Pearson’s or Spearman’s correlation coefficients

**Table 5:** Multivariate analysis of risk factors for increased contact pressure after additional coronal correction

Radiographic parameters	Hips with decreased CP (*n* = 15)	Hip with increased CP (*n* = 29)	Univariate *p* value	Multivariate *p* value
Lateral center-edge angle (°) ^a^	14.2 (−0.7 to 16.2)	7.5 (−14.8 to 17.4)	0.016	0.227
Medial center-edge angle (°) ^a^	46.3 ± 7.1	50.8 ± 9.5	0.104	
Acetabular arc (°) ^a^	59.6 (41.2–72.3)	58.2 (40.4–85.0)	0.742	
Tönnis angle (°) ^a^	21.3 (10.6–26.4)	23.0 (11.8–34.7)	0.102	
Sharp angle (°) ^a^	46.8 ± 3.7	48.4 ± 3.3	0.148	
Extrusion index (%) ^a^	33.3 (27.1–47.8)	41.1 (28.6–63.8)	0.002	0.030
Crossover sign ^b^	2 (13.3)	11 (37.9)	0.077	
Posterior wall sign ^b^	11 (73.3)	22 (75.9)	0.855	
Anterior wall index ^a^	28.3 ± 5.6	26.0 ± 14.2	0.531	
Posterior wall index ^a^	90.8 ± 16.2	85.4 ± 19.0	0.339	
Roundness index (%) ^a^	51.1 (48.9–56.6)	52.7 (49.3–57.6)	0.005	0.038
Femoro-epiphyseal acetabular roof index ^a^	6.4 (−1.5 to 12.8)	4.3 (−1.5 to 20.9)	0.457	

*CP* contact pressure

^a^Values are presented as the mean ± SD or the median (range)

^b^Values are presented as a number (%)

### Risk factors for abnormal contact pressure after correction

**Fig. 4:**
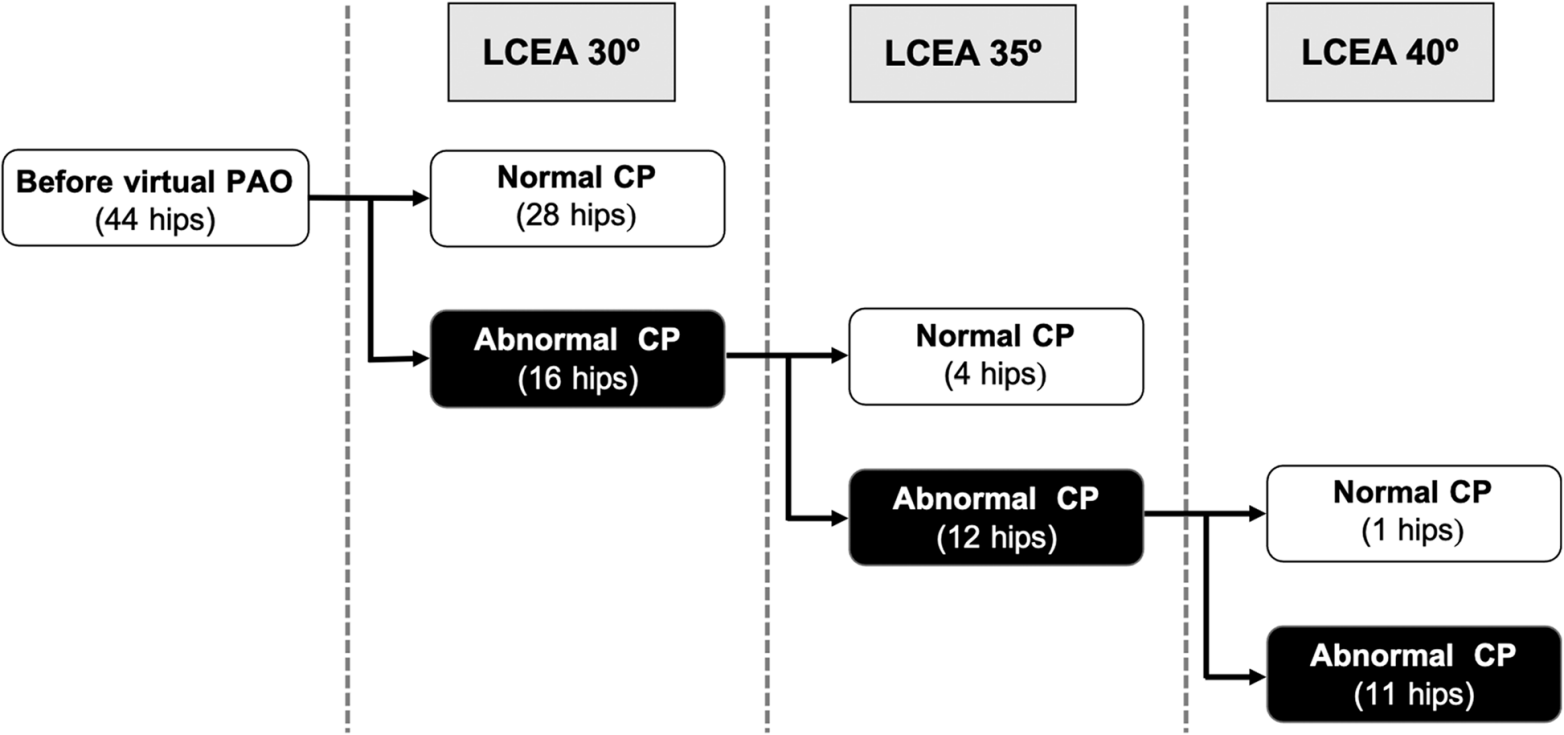
This flowchart shows stepwise acetabular correction in the coronal plane in virtual periacetabular osteotomy (PAO) and the corresponding number of hips within the normal range of the maximum joint contact pressure (CP) (< 4.1 MPa). Virtual PAOs normalized the maximum CP in a total of 33 hips (75%)

**Fig. 5:**
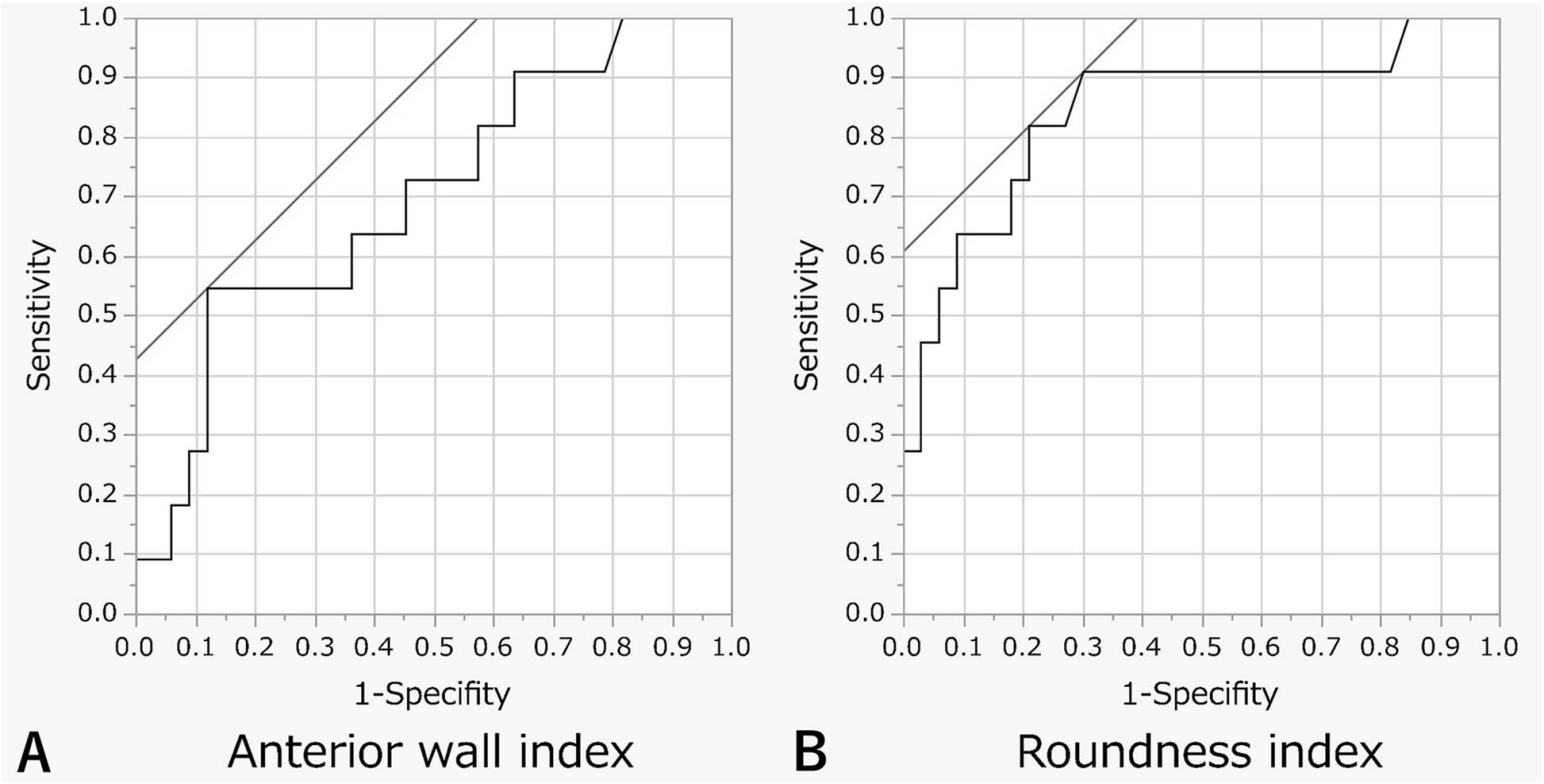
The receiver operating characteristic curves of the two independent morphological factors associated with abnormal maximum joint contact pressure after virtual periacetabular osteotomy. According to the curves, (**A**) the cut-off value of the anterior wall index was 0.21 (sensitivity 55%, specificity 88%, area under the curve [AUC] 0.77), and (**B**) that of the roundness index was 53.2% (sensitivity 82%, specificity 79%, AUC 0.84), on the preoperative standing pelvic radiographs

ROC curve analysis determined the cut-off value of the normal maximum CP as 4.1 MPa (sensitivity 100%, specificity 94%, area under the curve [AUC] 0.99). Based on this cut-off value, 28 hips (64%) achieved CP within the normal range at LCEA 30° (Table 3). When the stepwise correction was performed on 16 hips with residual abnormal CP, a correction of 5° (LCEA 35°) normalized the maximum CP in four hips (9%), and a further correction of 5° (LCEA 40°) normalized the maximum CP in one hip (2%) (Fig. 4). Thus, overall, the maximum CP was normalized in 33 hips (75%) following three patterns of PAO, while CP remained abnormal in 11 hips (25%). A multiple logistic regression analysis showed that a smaller preoperative acetabular wall index (*p* = 0.049) and a greater preoperative roundness index (*p* = 0.003) were independently associated with abnormal CP after virtual PAO (Table 6). ROC curve analysis determined that a preoperative anterior wall index < 0.21 (sensitivity 55%, specificity 88%, AUC 0.77) (Fig. 5A) and a roundness index > 53.2% (sensitivity 82%, specificity 79%, AUC 0.84) (Fig. 5B) on standing pelvic radiographs resulted in abnormal CP after virtual PAO.

**Table 6:** Multivariate analysis of risk factors for abnormal contact pressure after virtual periacetabular osteotomy

Radiographic parameters	Hips with normal CP (*n* = 33)	Hips with abnormal CP (*n* = 11)	Univariate *p* value	Multivariate *p* value
Lateral center-edge angle (°) ^a^	11.7 (−4.8 to 16.5)	3.3 (− 14.8 to 17.4)	0.023	0.388
Medial center-edge angle (°) ^a^	47.8 ± 8.6	53.7 ± 8.8	0.056	
Acetabular arc (°) ^a^	58.1 ± 8.7	59.0 ± 12.8	0.796	
Tönnis angle (°) ^a^	20.6 ± 5.2	24.6 ± 6.5	0.040	0.447
Sharp angle (°) ^a^	47.6 ± 3.4	48.4 ± 3.9	0.510	
Extrusion index (%) ^a^	37.0 ± 6.1	45.1 ± 11.3	0.006	0.503
Crossover sign ^b^	10 (30.3)	3 (27.3)	0.848	
Posterior wall sign ^b^	23 (69.7)	10 (90.9)	0.130	
Anterior wall index ^a^	29.0 ± 11.3	20.2 ± 11.8	0.033	0.049
Posterior wall index ^a^	90.9 ± 17.0	76.1 ± 17.4	0.014	0.098
Roundness index ^a^	51.8 ± 1.7	54.4 ± 2.3	< 0.001	0.003
Femoro-epiphyseal acetabular roof index ^a^	4.2 (−1.5 to 16.5)	4.3 (−1.5 to 20.9)	0.795	

*CP* contact pressure

^a^Values are presented as the mean ± SD or the median (range)

^b^Values are presented as a number (%)

## Discussion

Radiographic metrics such as LCEA and Tönnis angle are the most common parameters used to assess acetabular reorientation by PAO [4, 5, 8]. The mean LCEA after PAO varied among clinical studies, ranging from 24 to 41° [4–8, 14]. Several studies have shown that the clinical outcome of PAO was optimized by adjusting the LCEA between 30° and 40° [5, 9]. Wells et al. [7] showed a higher risk of PAO failure was associated with excessive femoral head coverage (LCEA > 38°). However, it remains unclear what the ideal acetabular position should be for each patient to optimize long-term joint survivorship following PAO. Kralj et al. [30] suggested that postoperative peak contact hip stress could be a better predictor of clinical outcome of PAO than the LCEA alone and is a useful tool to improve surgical planning. Therefore, we investigated the relationship between acetabular correction and CP via virtual PAO with LCEA between 30° and 40° to identify metrics for customizing acetabular reorientation according to individual hip joint morphologies.

In the present study, virtual PAO normalized CP in 64% of patients by correction to LCEA 30° and in 75% of patients by further corrections to LCEA 35° or 40°. Consistent with these results, previous studies have shown that coronal plane correction decreased joint CP using patient-specific FE models [10, 11]. Our results also suggested that the optimal position differed between patients: Joint CP was smallest in 55% of hips at LCEA 30°, in 25% at LCEA 35°, and in 20% at LCEA 40°. Three-dimensional hip morphology reportedly varies widely between PAO candidates; thus, it may be necessary to tailor the amount of acetabular correction to each patient rather than making a uniform correction [11–13]. In this study, the maximum CP increased in 66% of hips following correction from LCEA 30° to 40°, and this increase in CP was associated with greater preoperative extrusion index and roundness index. Okano et al. [31] reported a correlation between femoral head deformity and the severity of acetabular dysplasia and suggested that the femoral head’s shape may be influenced by the development of the acetabular weight-bearing area. An elliptical femoral head could lead to joint incongruity after acetabular correction and may adversely affect clinical outcomes after PAO [15, 16]. Therefore, in patients with a greater preoperative extrusion index and an aspherical femoral head, coronal plane correction beyond LCEA 30° may be ineffective in reducing CP. However, we performed virtual PAO without considering dynamic hip instability, and further studies are needed to clarify the impact of dynamic instability on the joint biomechanics of dysplastic hips and more comprehensively identify patient-specific targets for optimal acetabular correction in PAO.

In the current study, virtual PAO failed to normalize CP in 25% of patients, suggesting that there is a subgroup of patients whose CP cannot be normalized by coronal correction alone, even if the LCEA is corrected to the normal range. Specifically, in patients with insufficient anterior coverage (anterior wall index < 0.21) or an aspherical femoral head (roundness index > 53.2%), coronal plane correction alone may not be sufficient to normalize joint CP. Iwamoto et al. [25] reported that 19% of dysplastic hips had residual anterolateral acetabular deficiency after coronal plane correction with simulated PAO and that a preoperative anterior center-edge angle < 37° predicted residual deficiency. Stetzelberger et al. [32]. reported that a low anterior wall index was associated with conversion total hip arthroplasty in the long term after PAO. A theoretical model study demonstrated that anterolateral rotation of the acetabular fragment was more effective in reducing CP than lateral rotation alone [33]. Therefore, future studies should explore the effectiveness of multiplanar acetabular correction in improving the hip joint contact mechanism.

Previous studies have also shown that dysplastic hips often present with an elliptical femoral head and decreased head-neck offset, and that an aspherical femoral head could lead to joint incongruity after acetabular correction and may adversely affect clinical outcomes after PAO [15, 16]. Therefore, in cases with an elliptical femoral head, it is advisable to carefully simulate the joint conformity after POA via preoperative radiographs taken with the hip abducted.

Several limitations warrant discussion. First, we did not perform modeling of patient-specific cartilage or the labrum. Previous studies showed the similarity of peak CP between constant thickness cartilage (1.8 mm) models and patient-specific cartilage models [10]. Regarding validation of FE models without the labrum, Anderson et al. reported that subject-specific FE modeling of the hip joint without a labrum produced reasonable predictions of cartilage contact pressures and contact areas when compared directly to pressure film measurements [34]. Regarding validation of experimental load testing without a labrum, Konrath et al. reported that no significant differences were detected concerning contact pressure and contact area with or without a labrum under the single-leg stance using fresh frozen cadaver specimens [35]. It has been reported that the labrum plays a subsistent role in load transfer and joint stability [36]. However, it is unknown how labrum tears and cartilage wear affect its role in joint mechanics. Further studies are needed to clarify the effect of labrum and patient-specific cartilage on joint CP under conditions that incorporate actual labrum tears and cartilage wear. Second, we applied only static loading with constant joint resultant force, assuming one-leg stance without considering hip joint instability. Maeyama et al. [2]. demonstrated that PAO reduces dynamic instability in hip dysplasia; thus, we assumed that static loading was acceptable when using the FE model of virtual PAO. Loading conditions applied in this study were derived from in vivo data from patients who underwent THA [29] and are considered to approximate the actual loading conditions in the native hip. However, other conditions corresponding to daily activities and gait cycles were not assessed, and further studies are needed to clarify the impact of patient-specific joint resultant forces and dynamic instability on the joint biomechanics of dysplastic hips in other activities. Third, we used CT data with the slice thickness utilized in clinical practice, which may have resulted in inaccurate modeling. However, previous studies have validated FE analysis using CT data with a slice thickness of 2 or 3 mm for the hip joint using the same software as in this study [37, 38]. Therefore, our study using CT data with a slice thickness of 1 or 2 mm is considered valid. Finally, there is a possibility of inter-individual error in the model generation. However, the errors are expected to be small since all the model generation and FE analyses were performed by a single author in a consistent manner.

## Conclusions

The results of virtual PAO suggested that the degree of acetabular correction in the coronal plane where CP is minimized varied among patients. Coronal plane correction alone failed to normalize CP in 25% of patients in this study, and these patients had the morphological features of anterior acetabular deficiency and an aspherical femoral head. Specifically, in patients with an anterior wall index < 0.21 and a roundness index > 53.2% on standing pelvic radiographs, coronal plane correction alone may not normalize the hip joint biomechanics. Further studies in biomechanics-based planning for PAO should explore the impact of multiplanar acetabular correction, including sagittal and axial corrections, on the joint contact mechanics of dysplastic hips and its association with the clinical outcomes of PAO.

## Data Availability

The datasets used and/or analyzed during the current study are available from the corresponding author on reasonable request.

## Abbreviations

PAO: Periacetabular osteotomy
CP: Contact pressure
LCEA: Lateral center-edge angle
3D: Three-dimensional
OA: Osteoarthritis
FE: Finite-element
APP: Anterior pelvic plane
TOA: Transposition osteotomy of the acetabulum
AP: Anteroposterior
MCEA: Medial center-edge angle
HU: Hounsfield unit
ROC: Receiver operating characteristic
AUC: Area under the curve

## References

[1] Clohisy JC, Schutz AL, St John L, Schoenecker PL, Wright RW (2009). Periacetabular osteotomy: a systematic literature review. Clin Orthop Relat Res.

[2] Maeyama A, Naito M, Moriyama S, Yoshimura I (2009). Periacetabular osteotomy reduces the dynamic instability of dysplastic hips. J Bone Joint Surg (Br).

[3] Ganz R, Klaue K, Vinh TS, Mast JW (1988). A new periacetabular osteotomy for the treatment of hip dysplasias. Technique and preliminary results. Clin Orthop Relat Res.

[4] Grammatopoulos G, Wales J, Kothari A, Gill HS, Wainwright A, Theologis T (2016). What is the early/mid-term survivorship and functional outcome after Bernese periacetabular osteotomy in a pediatric surgeon practice?. Clin Orthop Relat Res.

[5] Hartig-Andreasen C, Troelsen A, Thillemann TM, Søballe K (2012). What factors predict failure 4 to 12 years after periacetabular osteotomy?. Clin Orthop Relat Res.

[6] Lerch TD, Steppacher SD, Liechti EF, Tannast M, Siebenrock KA (2017). One-third of hips after periacetabular osteotomy survive 30 years with good clinical results, no progression of arthritis, or conversion to THA. Clin Orthop Relat Res.

[7] Wells J, Schoenecker P, Duncan S, Goss CW, Thomason K, Clohisy JC (2018). Intermediate-term hip survivorship and patient-reported outcomes of periacetabular osteotomy: the Washington university experience. J Bone Joint Surg Am.

[8] Wyles CC, Vargas JS, Heidenreich MJ, Mara KC, Peters CL, Clohisy JC (2020). Hitting the target: natural history of the hip based on achieving an acetabular safe zone following periacetabular osteotomy. J Bone Joint Surg Am.

[9] Troelsen A, Elmengaard B, Soballe K (2008). A new minimally invasive transsartorial approach for periacetabular osteotomy. J Bone Joint Surg Am.

[10] Liu L, Ecker TM, Schumann S, Siebenrock KA, Zheng G (2016). Evaluation of constant thickness cartilage models vs. patient specific cartilage models for an optimized computer-assisted planning of periacetabular osteotomy. PLoS One.

[11] Zou Z, Chávez-Arreola A, Mandal P, Board TN, Alonso-Rasgado T (2013). Optimization of the position of the acetabulum in a Ganz periacetabular osteotomy by finite element analysis. J Orthop Res.

[12] Fujii M, Nakashima Y, Sato T, Akiyama M, Iwamoto Y (2011). Pelvic deformity influences acetabular version and coverage in hip dysplasia. Clin Orthop Relat Res.

[13] Nepple JJ, Wells J, Ross JR, Bedi A, Schoenecker PL, Clohisy JC (2017). Three patterns of acetabular deficiency are common in young adult patients with acetabular dysplasia. Clin Orthop Relat Res.

[14] Troelsen A, Elmengaard B, Søballe K (2009). Medium-term outcome of periacetabular osteotomy and predictors of conversion to total hip replacement. J Bone Joint Surg Am.

[15] Okano K, Enomoto H, Osaki M, Shindo H (2008). Rotational acetabular osteotomy for advanced osteoarthritis secondary to developmental dysplasia of the hip. J Bone Joint Surg (Br).

[16] Steppacher SD, Tannast M, Werlen S, Siebenrock KA (2008). Femoral morphology differs between deficient and excessive acetabular coverage. Clin Orthop Relat Res.

[17] Kitamura K, Fujii M, Ikemura S, Hamai S, Motomura G, Nakashima Y (2021). Does patient-specific functional pelvic tilt affect joint contact pressure in hip dysplasia? A finite-element analysis study. Clin Orthop Relat Res.

[18] Fujii M, Nakashima Y, Noguchi Y, Yamamoto T, Mawatari T, Motomura G (2011). Effect of intra-articular lesions on the outcome of periacetabular osteotomy in patients with symptomatic hip dysplasia. J Bone Joint Surg (Br).

[19] Wiberg G. Studies on dysplastic acetabula and congenital subluxation of the hip joint: with special reference to the complication of osteo-arthritis. Acta Chir Scand. 1939;83:5–135.

[20] Tönnis D, Legal H (1987). Congenital dysplasia and dislocation of the hip in children and adults.

[21] Siebenrock KA, Kistler L, Schwab JM, Büchler L, Tannast M (2012). The acetabular wall index for assessing anteroposterior femoral head coverage in symptomatic patients. Clin Orthop Relat Res.

[22] Tannast M, Hanke MS, Zheng G, Steppacher SD, Siebenrock KA (2015). What are the radiographic reference values for acetabular under- and overcoverage?. Clin Orthop Relat Res.

[23] Tachibana T, Fujii M, Kitamura K, Nakamura T, Nakashima Y (2019). Does acetabular coverage vary between the supine and standing positions in patients with hip dysplasia?. Clin Orthop Relat Res.

[24] Kitamura K, Fujii M, Utsunomiya T, Iwamoto M, Ikemura S, Hamai S (2020). Effect of sagittal pelvic tilt on joint stress distribution in hip dysplasia: a finite element analysis. Clin Biomech (Bristol, Avon).

[25] Iwamoto M, Fujii M, Komiyama K, Sakemi Y, Shiomoto K, Kitamura K (2020). Is lateral acetabular rotation sufficient to correct anterolateral deficiency in periacetabular reorientation osteotomy? A CT-based simulation study. J Orthop Sci.

[26] Keyak JH, Rossi SA, Jones KA, Skinner HB (1998). Prediction of femoral fracture load using automated finite element modeling. J Biomech.

[27] Wu G, Siegler S, Allard P, Kirtley C, Leardini A, Rosenbaum D, et al. ISB recommendation on definitions of joint coordinate system of various joints for the reporting of human joint motion--part I: ankle, hip, and spine. Int Soc Biomech J Biomech. 2002;35(4):543–8. 10.1016/s0021-9290(01)00222-6. 10.1016/s0021-9290(01)00222-6 11934426

[28] Caligaris M, Ateshian GA (2008). Effects of sustained interstitial fluid pressurization under migrating contact area, and boundary lubrication by synovial fluid, on cartilage friction. Osteoarthr Cartil.

[29] Bergmann G, Deuretzbacher G, Heller M, Graichen F, Rohlmann A, Strauss J (2001). Hip contact forces and gait patterns from routine activities. J Biomech.

[30] Kralj M, Mavčič B, Antolič V, Iglič A, Kralj-Iglič V (2005). The Bernese periacetabular osteotomy: clinical, radiographic and mechanical 7-15-year follow-up of 26 hips. Acta Orthop.

[31] Okano K, Yamaguchi K, Ninomiya Y, Matsubayashi S, Osaki M, Takahashi K (2013). Femoral head deformity and severity of acetabular dysplasia of the hip. Bone Joint J.

[32] Stetzelberger VM, Leibold CS, Steppacher SD, Schwab JM, Siebenrock KA, Tannast M (2021). The acetabular wall index is associated with long-term conversion to THA after PAO. Clin Orthop Relat Res.

[33] Hipp JA, Sugano N, Millis MB, Murphy SB (1999). Planning acetabular redirection osteotomies based on joint contact pressures. Clin Orthop Relat Res.

[34] Anderson AE, Ellis BJ, Maas SA, Peters CL, Weiss JA (2008). Validation of finite element predictions of cartilage contact pressure in the human hip joint. J Biomech Eng.

[35] Konrath GA, Hamel AJ, Olson SA, Bay B, Sharkey NA (1998). The role of the acetabular labrum and the transverse acetabular ligament in load transmission in the hip. J Bone Joint Surg Am.

[36] Henak CR, Ellis BJ, Harris MD, Anderson AE, Peters CL, Weiss JA (2011). Role of the acetabular labrum in load support across the hip joint. J Biomech.

[37] Utsunomiya T, Motomura G, Ikemura S, Kubo Y, Sonoda K, Hatanaka H (2018). Effects of sclerotic changes on stress concentration in early-stage osteonecrosis: a patient-specific, 3D finite element analysis. J Orthop Res.

[38] Bessho M, Ohnishi I, Matsuyama J, Matsumoto T, Imai K, Nakamura K (2007). Prediction of strength and strain of the proximal femur by a CT-based finite element method. J Biomech.

